# Urine-Derived Renal Epithelial Cells (URECs) from Transplanted Kidneys as a Promising Immunomodulatory Cell Population

**DOI:** 10.3390/cells12121630

**Published:** 2023-06-15

**Authors:** Valeria Pizzuti, Chiara Donadei, Emma Balducelli, Diletta Conte, Elisa Gessaroli, Francesca Paris, Claudia Bini, Marcello Demetri, Miriam Di Nunzio, Valeria Corradetti, Francesco Alviano, Gaetano La Manna, Giorgia Comai

**Affiliations:** 1 Department of Medical and Surgical Sciences (DIMEC), University of Bologna, 40126 Bologna, Italy; valeria.pizzuti3@unibo.it (V.P.); emma.balducelli2@unibo.it (E.B.); diletta.conte2@unibo.it (D.C.); elisa.gessaroli4@studio.unibo.it (E.G.); francesca.paris6@unibo.it (F.P.); marcello.demetri2@unibo.it (M.D.); miriam.dinunzio@studio.unibo.it (M.D.N.); 2 Nephrology, Dialysis and Renal Transplant Unit, IRCCS Azienda Ospedaliero-Universitaria di Bologna, 40139 Bologna, Italy; chiara.donadei@studio.unibo.it (C.D.); claudia.bini@aosp.bo.it (C.B.); valeria.corradetti@aosp.bo.it (V.C.); giorgia.comai@aosp.bo.it (G.C.); 3 Department of Biomedical and Neuromotor Sciences (DIBINEM), University of Bologna, 40126 Bologna, Italy; francesco.alviano@unibo.it

**Keywords:** chronic kidney disease, kidney transplant, ischaemia-reperfusion, acute kidney injury, urine cells, urine-derived renal epithelial cells, kidney progenitor cells, immunomodulatory capacity, lymphocytes, T regulatory cells

## Abstract

Kidney transplantation is a lifesaving procedure for patients with end-stage kidney disease (ESKD). Organs derived from donation after cardiac death (DCD) are constantly increasing; however, DCD often leads to ischaemia-reperfusion (IR) and Acute Kidney Injury (AKI) events. These phenomena increase kidney cell turnover to replace damaged cells, which are voided in urine. Urine-derived renal epithelial cells (URECs) are rarely present in the urine of healthy subjects, and their loss has been associated with several kidney disorders. The present study aimed to characterize the phenotype and potential applications of URECs voided after transplant. The results indicate that URECs are highly proliferating cells, expressing several kidney markers, including markers of kidney epithelial progenitor cells. Since the regulation of the immune response is crucial in organ transplantation and new immunoregulatory strategies are needed, UREC immunomodulatory properties were investigated. Co-culture with peripheral blood mononuclear cells (PBMCs) revealed that URECs reduced PBMC apoptosis, inhibited lymphocyte proliferation, increased T regulatory (Treg) cells and reduced T helper 1 (Th1) cells. URECs from transplanted patients represent a promising cell source for the investigation of regenerative processes occurring in kidneys, and for cell-therapy applications based on the regulation of the immune response.

## 1. Introduction

Kidney transplantation is the treatment of choice for the resolution of chronic kidney disease (CKD) and end-stage kidney disease (ESKD), conferring a significant improvement to the quality of life and an increase in survival rate compared with dialysis [1] (pp. 2093–2109). As with many other surgeries, several complications can arise because of the transplant procedure, including nephrotoxicity and ischaemia-reperfusion (IR) damage [2] (pp. 1546–1549). The IR process represents an inevitable event in the early phases of kidney transplantation, and it is associated with the onset of acute kidney injury (AKI), delayed graft function (DGF), along with an increased risk of graft rejection. This process is linked to the impairment of tubular-cell morphology and functionality, along with alterations in the cytokine microenvironment and in tissue homeostasis [3] (p. 1412).

These events result in an increased cell turnover within nephron structures, with the recruitment of renal progenitor cells that replace damaged cells and contribute to the regeneration of kidney structures [4] (p. 251). The result of the regeneration process is an increased exfoliation of epithelial cells lining tubules, which are highly voided in the urine [5] (p. 534139).

Different cell types of the urinary tract are physiologically voided into the urine daily; however, only negligible amounts of kidney cells have been isolated from the urine of healthy subjects. On the contrary, an increase in the exfoliation rate has been observed in active kidney diseases, including AKI and CKD [6,7] (pp. 525–531, p. 7610). In this context, urine may represent a promising, easily obtainable and ethics-free cell source for diagnostic and therapeutic applications, avoiding the invasiveness and the risks of solid biopsies [8].

Numerous cell-isolation and -characterization techniques have been developed in recent years, allowing exfoliated cells from the urine to be used as surrogate markers for solid biopsies in predicting changes relating to gene expression, deoxyribonucleic acid (DNA) methylation, DNA damage and protein expression in the kidney [9] (pp. 601–610). In addition to their application in diagnostics, urine cells are increasingly studied for cell therapy applications in different pathologies, as well as in drug-testing procedures [10] (p. 189). Since the applications of exfoliated kidney cells into clinical practice has increased, the development of protocols to properly isolate and expand the target-cell type, and the set-up of proper culture conditions are required [11] (pp. 345–349).

As of today, the cell types commonly observed and isolated in the urine of patients with different pathological conditions include podocytes, tubular progenitor cells and proximal tubule epithelial cells (PTECs) [12] (pp. F145–F161). Urine is also a source of cells with stemness characteristics, and immunomodulatory and differentiative abilities [13,14] (pp. 3566–3578, p. 573); these cells are known as urine stem cells (USCs) and share some phenotypical and functional features with mesenchymal Stem Cells (MSCs), derived from other tissues [10,11] (p. 189, pp. 345–349). Unlike embryonic stem cells (ESCs) USCs can be transplanted into an animal model without inducing teratoma formation [15,16] (pp. 2123–2132, pp. 1–20). Given the high heterogeneity of the urine sediment, the kidney epithelial populations obtained during culture are commonly defined as urine-derived renal epithelial cells (URECs) [17] (pp. 87–95).

URECs are another population of progenitor cells found in urine, considered to be more differentiated and less proliferating than USCs. URECs can be isolated by the centrifugation of urine samples, and the seeding of cell suspension under proper conditions allows one to subculture cells for 4 to 10 passages, before they visibly transform, acquiring a more flattened and elongated morphology, suggesting the onset of senescence. These cells share most of their markers with USCs, differing in terms of their morphology and differentiation potential. In fact, URECs’ phenotype is intermediate between PTECs and fibroblasts, with the expression of cytokeratin and proximal-tubule-associated markers, such as CD13 [18] (pp. 1–17), along with the expression of fibroblastic-like markers such as CD90 and Vimentin [17] (pp. 87–95).

There are certain pathological conditions in which URECs are highly voided in urine, but a small amount of progenitor epithelial cells was also found in urine samples from preterm neonates [19] (pp. 2762–2770). A loss of renal tubular epithelial cells was also observed in the urinary sediments of renal transplant recipients, especially in the first 2 weeks post-kidney-transplant, with an increase in their number during acute graft rejection, as demonstrated in a pivotal study published in 1977 [20] (pp. 580–584). The primary objective of the present study is to isolate and characterize URECs obtained from patients who have undergone kidney transplants, focusing on evaluating their phenotype and proliferative capacity. Moreover, the search for cells with immunomodulatory and anti-inflammatory capacities is one of the most attractive fields in regenerative medicine strategies; here, for the first time, we accurately describe the ability of URECs to reduce CD4 T (CD4^+^) and CD8 T (CD8^+^) lymphocyte proliferation, promoting the expansion of regulatory T cells (Treg), while reducing the T helper 1 (Th1)-producing interferon gamma (IFN-γ), and modulating the cytokine microenvironment.

## 2. Materials and Methods

### 2.1. Patients Recruitment and Literature Search

The urine samples collected for this study were derived from patients who had undergone kidney transplantation in University Hospital of Bologna IRCCS, Sant’Orsola Polyclinic, after the approval of the Ethics Committee (protocol number: 312/2021/Oss/AOUBO).

The transplanted kidneys were derived from circulatory death (DCD) donors. Transplanted patients recruited for the study had no residual diuresis, to ensure the donor origin of kidney cells. The urine of four healthy volunteers was collected as the control. Information from the literature was selected, starting from papers regarding differences about kidney donors, focusing on DCD ones. The side effects of DCD donation on kidneys were also evaluated, followed by a search for studies regarding urine-derived kidney cells and their applications. Subsequently, the study of the literature regarding the immunomodulatory properties of different cell populations was performed.

### 2.2. Isolation of Urine Renal Epithelial Cells (URECs)

The urine of transplant patients was collected within the first week after transplantation (T0), when patients resume autonomous diuresis. Urine samples were also collected after one (T1) and six (T6) months from kidney transplant. Samples were processed right after the collection and the range of urine volume was 100–300 mL. Urine was transferred into sterile 50 mL tubes and centrifuged at 400 g for 10 min. The pellet was resuspended in phosphate-buffered saline (PBS, Corning, Steuben County, NY, USA) with 1% penicillin–streptomycin (P/S) solution (10,000 U/mL Penicillin, 10,000 U/mL Streptomycin, Corning, Steuben County, NY, USA) and centrifuged at 400 g for 10 min. Urine sediment was resuspended in Dulbecco’s modified Eagle medium: Nutrient Mixture F12 (DMEM F12, Gibco, Life Technologies, Carlsbad, CA, USA) with 10% of foetal bovine serum (FBS, Gibco, Life Technologies, Carlsbad, CA, USA), and supplemented with Renal Epithelial Growth Medium (REGM^TM^) Single Quot kit (Lonza Bioscience, Basel, Swizerland), 1% P/S solution (Corning, Steuben County, NY, USA), 2.5 µg/mL amphotericin B (Biochrom, Waterbeach Cambridge, UK), 100 µg/mL normocin (InvivoGen, San Diego, CA, USA) and 10 µg/mL ciprofloxacin (Fresenius Kabi, Graz, Austria).

The cell suspension was seeded in flasks and incubated at 37 °C and 5% CO_2_. After 72 h, the isolation medium was replaced with Renal Epithelial Basal Medium (REBM^TM^, Lonza Bioscience, Basel, Swizerland) at 10% of FBS and supplemented with REGM ^TM^ SingleQuot kit, 2.5 mM GlutaMAX (Gibco, Life Technologies, Carlsbad, CA, USA), 1% non-essential amino acids (Termo Fisher Scientific, Waltham, MA, USA) and 1% P/S. The proliferation medium allows the survival and proliferation of epithelial cells, while dead cells and cellular debris are removed with subsequent medium exchanges. Cultures were monitored for the presence of growth foci, and after reaching confluence, the cells were detached from the growth surface by incubation with 0.25% Trypsin/EDTA solution (Corning, Steuben County, NY, USA). For each time point, the isolation yield percentage in the Y axes refers to the number of patients in which UREC cells could be isolated and grown in culture. The freshly isolated cells were counted and tested for viability using Erythrosine B (Sigma-Aldrich, St. Louis, MO, USA). Only samples with >90% viability were used for further assays. All the experiments were performed on cells obtained at T0 and used within the third culture passage.

### 2.3. Immunofluorescence Analysis

For immunofluorescence analysis, URECs were seeded onto glass coverslips and cultured in proliferation medium. Cells were fixed with 10% formalin for ten minutes at room temperature, washed with PBS, and permeabilized by adding PBS 0.1% Triton (Triton X-100, Sigma-Aldrich, Co., St. Louis, MO, USA) for 10 min. URECs were incubated for 30 min with blocking solution containing PBS 1% Bovine Serum Albumin (BSA, Sigma-Aldrich, St. Louis, MO, USA) and then incubated overnight at 4 °C with primary antibodies rabbit anti-Vimentin (1:200, Thermo Fisher Scientific, Waltham, MA, USA) and mouse anti-Ki-67 FITC (1:250, Miltenyi-Biotech, Bergisch Gladbach, NRW, Germany), and mouse anti-CD24 (1:250, #MAB8547, R&D Systems, Minneapolis, MN, USA) diluted in blocking solution. Secondary antibodies anti-rabbit Alexa Fluor 594 (1:500, Thermo Fisher Scientific, Waltham, MA, USA) and anti-mouse Alexa Fluor 488 (1:500, #A11001, Thermo Scientific, Waltham, MA, USA) were added and incubated for 1 h at room temperature. After three washes with PBS, coverslips were mounted using the Prolong Gold Antifade Mountant with DAPI (Thermo Fisher Scientific, Monza, Italy). Stained cells were observed using a Nikon Inverted Microscope (Nikon Instruments, Tokyo, Japan), and images were acquired with a Digital Sight camera DS-03 using the imaging software NIS-Elements 4.1 (Nikon Corporation, Tokyo, Japan).

### 2.4. URECs Proliferation during Culture Passages

To assess the proliferation of UREC during culture passages, cells were thawed and seeded in proliferation medium. The proliferative capacity of URECs after each passage (P1–P5) was assessed as Cumulative Population Doubling (CPD) by applying the following formula: [log10(NH) − log10(N1)]/log10(2)], where NH is the number of harvest cells, and N1 is the number of plated cells.

### 2.5. RNA Extraction and Real-Time PCR

URECs were seeded in T25 flasks at the density of 30,000 cells/cm^2^ and cultured in proliferation medium. For the extraction of RNA, from UREC and PBMC samples, the RNeasy mini kit (QIAGEN, Valencia, CA, USA) was used following the manufacturer’s instructions. The genomic DNA contamination was removed by digestion with RNase-free deoxyribonuclease I (RNase-free DNase set, QIAGEN, Valencia, CA, USA). The evaluation of RNA quality and concentration was assessed using the NanoDrop^®^ 1000 Spectrophotometer (Thermo Fisher Scientific, Waltham, MA, USA). The iScript cDNA Synthesis Kit (Bio-Rad Laboratories, Hercules, CA, USA) was used to reverse-transcribe the RNA according to the manufacturer’s instructions. Real-time PCR (qPCR) was performed in a Bio-Rad CFX96 real-time thermal cycler (Bio-Rad Laboratories, Hercules, CA, USA). For each condition, 25 ng of cDNA were amplified using the SsoAdvanced Universal SYBR Green Supermix (Bio-Rad Laboratories, Hercules, CA, USA) in technical triplicate. Data were analysed using the software CFX Manager (Bio-Rad Laboratories, Hercules, CA, USA) and the 2−ΔΔCt method. The glyceraldehyde-3-phosphate dehydrogenase (GAPDH) was used as the reference gene and the expression of CD24 and CD133 was normalized on GAPDH. The primers used were as follows: GAPDH (Cat. No: HP205798, ID: NM_002046, Origene, Rockville, MD, USA), CD24 (Cat. No: HP210404, ID: HP210404, Origene, Rockville, MD, USA), CD133 (Cat. No: HP209042, ID: NM_006017, Origene, Rockville, MD, USA)

### 2.6. Flow Cytometry Analysis of URECs Immunophenotype

After isolation, URECs were seeded in culture flasks and cultured until confluence. Cells were harvested by trypsin digestion and fixed with 10% formalin (Sigma-Aldrich, St. Louis, MO, USA) for 10 min. Fixed cells were stained with conjugated antibodies and analysed by flow cytometry. For the surface staining, fixed cells were incubated for 30 min at 4 °C in staining buffer, made of PBS containing 0.1% BSA (Sigma-Aldrich, St. Louis, MO, USA), avoiding light exposure. Conjugated antibodies used for surface staining were the following: anti-CD13 APC, anti-CD326 (EpCam) FITC, anti-CD24 PE, anti-CD90 PerCP/Cy5.5, anti-CD133 APC, all purchased from BioLegend, San Diego, CA, USA, and anti-HLA-G PerCp-eFluor™ 710 (eBioscience, San Diego, CA, USA). Cells were then washed twice and resuspended in PBS 0.1% BSA. For the intracellular staining, cells were permeabilized using a Fixation/Permeabilization Kit (BD Bioscience, Franklin Lakes, NJ, USA) according to the manufacturer’s instructions. Cells were incubated for 30 min at 4 °C in permeabilization buffer with anti-Cytokeratin (Ck) BV421 (BD Bioscience, Franklin Lakes, NJ, USA) and anti Ki-67 FITC (Miltenyi-Biotech, Bergisch Gladbach, NRW, Germany) antibodies. Cells were resuspended in PBS 0.1% BSA and analysed by flow cytometry using a CytoFLEX S (Beckman Counter, CA, USA) instrument. Unstained samples were used as negative controls and the data were analysed using FlowJo X software V.10 (Tree Star, Ashland, OR, USA). The markers were analysed and their functions are listed in the table below (Table 1).

### 2.7. Isolation of PBMCs

PBMCs were obtained from the blood of healthy donors according to the protocol approved by the Ethics Committee. PBMCs were isolated by density gradient centrifugation with Histopaque^®^-1077 (Sigma-Aldrich, St. Louis, MO, USA). Then, the blood sample was centrifuged at 2500 rpm for 30 min without break. After the centrifugation, the ring containing the PBMC population was collected and resuspended in PBS. The cell suspension was centrifuged at 1500 rpm for 10 min, and the washing step was repeated twice. PBMCs were counted with Methyl Violet (Sigma-Aldrich, St. Louis, MO, USA) to exclude red blood cells and with Erythrosine B to evaluate viability; cells were frozen at –80 °C in FBS with 10% dimethyl sulfoxide (Sigma-Aldrich, St. Louis, MO, USA). 

### 2.8. Co-Culture of URECs and PBMCs

URECs were seeded at a density of 40,000 cells/cm^2^ in 96-well plates in proliferation medium. After 24 h, PBMCs were thawed and activated by stimulation with anti-CD3 (CD3 Monoclonal Antibody HIT3a, Functional Grade, eBioscience™ Invitrogen, Waltham, MA, USA) and anti-CD28 (CD28 Monoclonal Antibody (CD28.2), Functional Grade, eBioscience™, Invitrogen, Waltham, MA, USA) antibodies. Stimulated PBMCs were seeded at a concentration of 200,000 cells/well above the URECs monolayer (PBMCs + URECs); activated PBMCs seeded in the absence of URECs were set as the positive control, while non-activated PBMCs represented the negative control. Cells were incubated for 72 h, and the medium for the co-culture experiments was RPMI 10% FBS, 2.5 mM GlutaMAX (Gibco), 1% non-essential amino acids (Termo Fisher Scientific, Waltham, MA, USA) and 1% P/S. Proliferation, apoptosis, and analysis of lymphocyte subpopulations were assessed by flow cytometry.

### 2.9. CFSE Assay

The proliferation of CD4^+^ and CD8^+^ lymphocytes with or without co-culture with URECs was evaluated by labelling with Carboxyfluorescein Succinimidyl Ester (CFSE). Before seeding, PBMCs were labelled with BD Horizon™ CFSE according to the manufacturer’s protocol. Briefly, thawed PBMCs were resuspended in PBS and labelled with CFSE 2 µM for 5 min at 37 °C. The reaction was stopped using RPMI with 50% of FBS; cells were then centrifuged and resuspended in culture medium. CFSE-labelled PBMCs were activated with anti-CD3 and anti-CD28 antibodies and seeded at a concentration of 200,000 cells/well in a 96-well plate for both positive controls and co-culture condition, as previously described. After 72 h, PBMCs were collected, washed with PBS, and stained with anti-CD4 APC (Biolegend, San Diego, CA, USA) and anti-CD8 Pecy7 (Biolegend, San Diego, CA, USA) antibodies for 30 min at 4 °C in PBS 0.1% BSA, both diluted at 1:100. The proliferation rate of CD4^+^ and CD8^+^ cells was evaluated by flow cytometry using the CytoFLEX S instrument. Results were compared to positive control set as 100%. A total of 20,000 events among both CD4^+^ and CD8^+^ cell subsets were acquired for each sample.

### 2.10. Analysis of PBMCs Apoptosis with Annexin V/7-AAD Assay

The apoptosis of PBMCs with or without co-culture was assessed using Annexin V/7-AAD kit (Biolegend, San Diego, CA, USA). Cells were stained according to manufacturer instructions. Briefly, PBMCs were collected, washed with PBS, and then labelled for 15 min at room temperature with anti-Annexin V PE and 7-AAD in binding buffer; both used 2:100. After the staining, 400 µL of binding buffer was added to each tube and samples were analysed by flow cytometry. Unstained PBMCs were used as negative controls and results were represented as a percentage of Annexin V PE^+^/7-AAD^−^ (early apoptosis) and Annexin V PE^+^/7-AAD^+^ (late apoptosis) cells among PBMCs.

### 2.11. Flow Cytomentry Analysis of Lymphocyte Subpopulations

PBMCs from healthy donors were cultured with URECs as described before. After 72 h of incubation, PBMCs were collected, and the characterization of T cells subset was performed. For the analysis of the Treg population, anti-CD4 APC (Biolegend, San Diego, CA, USA) and anti-CD25 FITC (Biolegend, San Diego, CA, USA) antibodies were used as surface markers, through incubation of 30 min in PBS 0.1% BSA. For intracellular staining, PBMCs were fixed and permeabilized with eBioscience™ Foxp3/Transcription Factor Staining Buffer Set (Invitrogen, MA, USA) according to the manufacturer’s instructions. Briefly, cells were fixed for 30 min at 4 °C with Fixation/Permeabilization solution, previously diluted 1:4 with Foxp3 Fixation/Permeabilization Diluent, and permeabilized with 1X Foxp3 Permeabilization Buffer for 15 min. Cells were labelled with anti-FoxP3 PE (Biolegend, San Diego, CA, USA) antibody for 30 min at 4 °C in Permeabilization Buffer, washed twice and resuspended in PBS 0.1% BSA. For the quantification of intracellular cytokines, the PBMCs of both the control and co-culture conditions were treated with a mixture of 1 nM Phorbol 12-myristate 13-acetate (PMA, Sigma-Aldrich, St. Louis, MO, USA), Ionomycin 3 mg/mL (Sigma-Aldrich, St. Louis, MO, USA) and 1 uL/mL Golgi Plug™ (BD, Becton, Dickinson, NJ, USA), and incubated at 37 °C to 5% CO_2_ for 4 h before staining. For IFN-γ-producing populations, cells were stained for surface markers with the anti-CD4 APC and (Biolegend, San Diego, CA, USA) antibody, while intracellular staining was performed, after the fixation and permeabilization steps, for 30 min at 4 °C with anti-IFN-γ-PeCy7 (Biolegend, San Diego, CA, USA). The markers for the identification of PBMC subsets and their functions are listed in Table 2.

### 2.12. Luminex xMAP Thecnology

The analysis of cytokine release was performed in cell culture supernatant of PBMCs and PBMCs + URECs conditions. After 72 h of culture, conditioned media were centrifuged and collected into 1.5 mL tubes; samples were stored at −80 °C until subsequent analysis. Samples were thawed at room temperature and cytokines were quantified using the Human Custom Procartaplex-19 (Cat. No. PPX-19-MXRWE2G, Invitrogen, Waltham, MA, USA) according to manufacturer protocol, and the concentration of cytokines was measured by MAGPIX TM (Luminex^®^ xMAP^®^ Technology, Austin, TX, USA). All samples were analysed in technical triplicates. The amount of each cytokine in the co-culture condition was normalized to the control condition (PBMCs), set as 100%. Table 2 reports the list of analysed cytokines.

### 2.13. Statistical Analysis

All the experiments were performed at least on five donors. Data are expressed as mean ± standard deviation (SD) and were analysed with a *t*-test using Graph Pad Prism 7.04 software (San Diego, CA, USA). The significance threshold was *p* < 0.05.

## 3. Results

### 3.1. Isolation of URECs Is Possible Post-Kidney-Transplantation at the Time of Resumption of Diuresis

**Figure 1:**
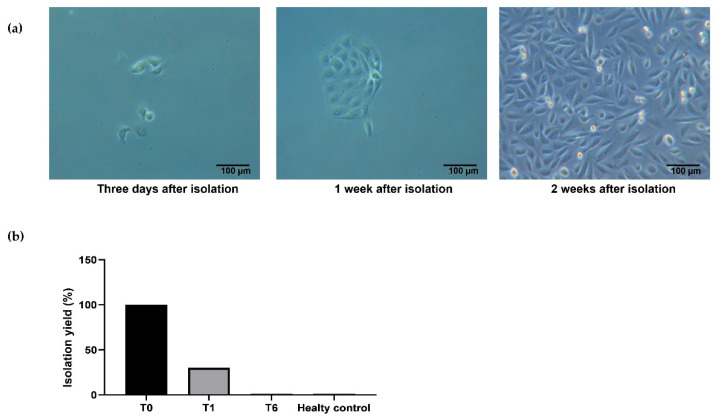
Isolation and culture of URECs: (**a**) representative images of URECs obtained after isolation. (**b**) Percentage of patients where UREC isolation and culture has been successful. Transplanted patients (n = 12 for each time point T0, T1, T6), healthy controls (n = 5). Isolation yield was evaluated shortly after transplant (T0 = 100%, n = 12), at one month (T1 = 30%, n = 4) and six months (T6 = 0%, n = 0) after surgery, and in healthy controls (n = 0).

Urine was firstly collected between 5–10 days after kidney transplant (T0); sample collection was also performed after one (T1) and six months (T6). In all the patients recruited for the study, the isolation of URECs at T0 was successful and cells reached confluence in two weeks (Figure 1a). The possibility to successfully isolate and culture URECs significantly decreased at time point T1. At time point T6, no successful isolations were observed from the patients recruited for the study, and the same result was obtained in healthy subjects (healthy control) (Figure 1b).

### 3.2. URECs Are Highly Proliferating Cells

**Figure 2:**
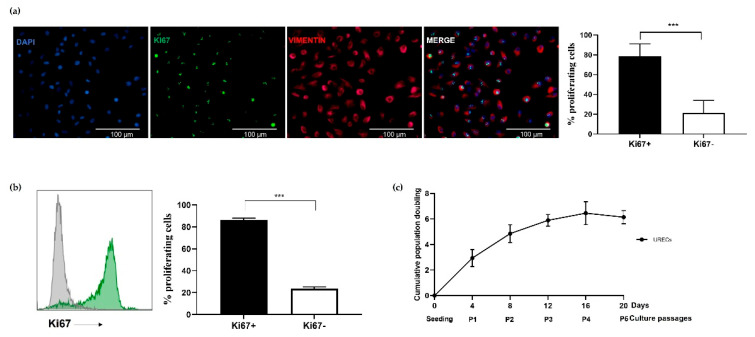
Assessment of URECs proliferative activity: (**a**) Immunofluorescence analysis of URECs’ proliferative activity. Images represent cells stained with DAPI (blue), Ki-67 (green) and Vimentin (red); the related graph shows the percentage of Ki-67^+^ (83.63 ± 12.53%) and Ki-67 − (21.40 ± 12.53%) cells. Magnification 40×, scale bar 100 µm. (**b**) Flow cytometry analysis for Ki-67^+^ (87 ± 6.74%) and Ki-67^−^ (23.63 ± 9.34%) cells and related graph. (**c**) Cumulative Population Doubling of URECs during culture passages (P1–P5). Results are expressed as mean ± SD, *** *p* < 0.001, n = 5 independent experiments.

The immunofluorescence analysis of Ki-67 nuclear expression on isolated URECs revealed the presence of two populations, allowing us to distinguish cells in active proliferation (Ki-67^+^) from less-proliferating cells (Ki-67^−^). As shown in the representative immunofluorescence images and in the related graph (Figure 2a), the percentage of Ki-67^+^ cells was significantly higher than the negative subset. Results obtained by immunofluorescence were also confirmed by flow cytometry (Figure 2b). The proliferative activity of URECs was maintained for up to four culture passages (Figure 2c).

### 3.3. URECs Are Proximal Tubule-Derived Epithelial Cells, Expressing Progenitor Cell, Mesenchymal Stromal Cell and Tolerogenic Markers

**Figure 3:**
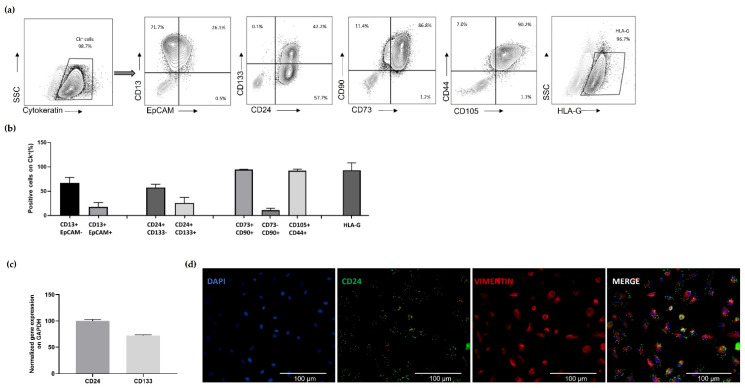
Analysis of URECs immunophenotype: (**a**) Flow cytometry gating strategy analysis of URECs phenotype and (**b**) histograms representing the percentage of positive cells, on Cytokeratin-positive (96.8 ± 7.04%), for each marker: CD13^+^EpCAM − (89.53 ± 12.48%), CD13^+^EpCAM^+^ (30 ± 12.8%), CD24^+^CD133 − (66.39 ± 5.6%), CD24^+^CD133^+^ (67.05 ± 14.3%),CD73^+^CD90^+^ (86.8 ± 3.75%), CD73 −CD90^+^ (11.4 ± 4.96%), CD44^+^CD105^+^ (90.2 ± 3.65%), HLA-G (96.7 ± 16.06%). (**c**) Real Time PCR for CD24 and CD133 gene expression; results were normalized on GAPDH. (**d**) Immunofluorescence evaluation of CD24; images represent cells stained with DAPI (blue), CD24 (green) and Vimentin (red), magnification 40×, scale bar 100 µm. Results are expressed as mean ± SD, n = 5 independent experiments.

The flow cytometry analysis (Figure 3a,b) of the immunophenotype of UREC revealed that cells were highly positive for the epithelial marker Cytokeratin (Ck^+^). Among Ck^+^ cells, a high expression of proximal tubule marker CD13 was observed, while the expression of the distal tubule marker Epithelial Cell Adhesion Molecule (EpCAM) was lower and majorly co-expressed with CD13. The expression of CD24 and CD133 markers, which are both known as typical surface molecules of kidney epithelial progenitor cells, was also evaluated. The analysis revealed the presence of two UREC subpopulations, a more represented CD24^+^CD133− and a less detected CD24^+^CD133^+^. URECs also express typical mesenchymal stromal cell markers, characterized for CD73^+/^−CD90^+^ and CD44^+^CD105^+^. Interestingly, URECs also expressed the tolerogenic molecule HLA-G. Real-time PCR and immunofluorescence analysis confirmed the high expression of CD24 and CD133 (Figure 3c,d).

### 3.4. URECs Reduced Proliferation of CD4^+^ and CD8^+^ Lymphocytes without Promoting PBMC Apoptosis

**Figure 4:**
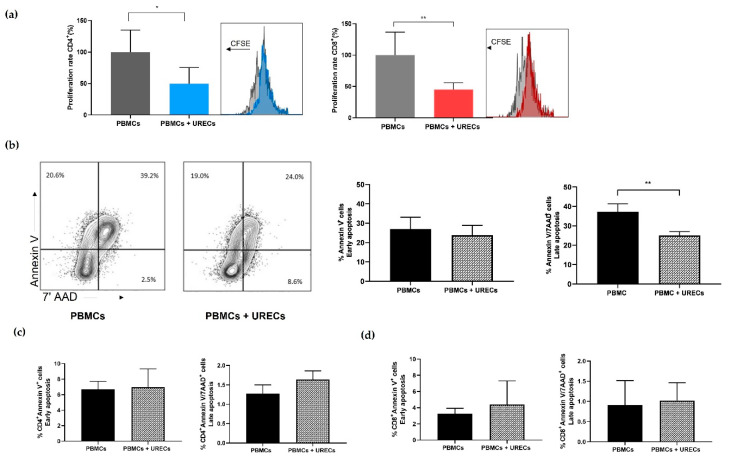
CD4^+^ and CD8^+^ cell-proliferation and -apoptosis assay during PBMC–UREC co-culture: (**a**) CFSE assay analysis of CD4^+^ (blue bar, 49.55 ± 25.8%) and CD8^+^ (red bar, 45.08% ± 10.72%) cell-proliferation rate in PBMCs co-cultured with URECs compared with control PBMCs (grey), set as 100%. (**b**) Annexin V/7-AAD assay for apoptosis of PBMCs. Histograms show the percentage of PBMCs in early (Annexin V^+^/7-AAD−) and late (Annexin V^+^/7-AAD^+^) apoptosis in PBMCs (Annexin V^+^/7-AAD−: 27.02 ± 6.13%; Annexin V^+^/7-AAD^+^: 23.85 ± 5%) and the PBMCs + URECs condition (Annexin V^+^/7-AAD−: 37.28 ± 4.05%; Annexin V^+^/7-AAD^+^: 25.05 ± 1.97%). (**c**,**d**) Annexin V/7-AAD assay for the apoptosis of CD4^+^ (**c**) and CD8^+^ (**d**) cells. Histograms show the percentage of CD4^+^ and CD8^+^ cells in early and late apoptosis in the PBMCs and PBMCs + URECs conditions. Results are expressed as mean ± SD, * *p* < 0.05, ** *p* < 0.01, n = 5 independent experiments.

The co-culture of URECs with anti-CD3- and anti-CD28-activated PBMCs was set up to analyse the interaction between these cell populations during in vitro culture and to evaluate the immunomodulatory capacity of kidney-derived cells. As shown in the pictures of Figure 4a, the co-culture (PBMCs + URECs) significantly reduced the activation of PBMCs compared to the positive control (PBMCs). Particularly, the proliferation of both CD4^+^ and CD8^+^ cells was significantly impaired in the co-culture condition when compared with activated PBMCs without URECs, set as control. To explain the reduction in PBMC proliferation when cultured with URECs, the analysis of early and late apoptosis was performed on PBMCs with and without co-culture, and results are represented in Figure 4b. While the number of cells in early apoptosis (Annexin V^+^/7-AAD−) remained stable, the percentage of cells undergoing late apoptosis (Annexin V^+^/7-AAD^+^) was significantly lower in co-culture conditions, compared to controls. The early and late apoptosis of CD4^+^ and CD8^+^ cells were not affected by the co-culture with URECs (Figure 4c,d)

### 3.5. URECs Increased the Percentage of Treg Cells, While Reducing the Th1 Subset

**Figure 5:**
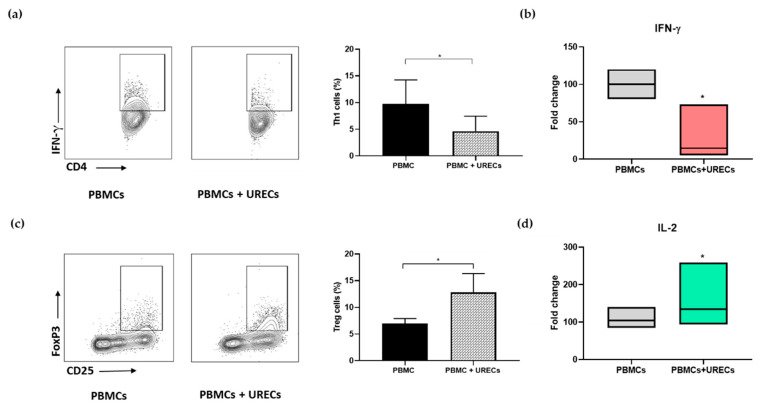
CD4^+^ cell subset and cytokine release during PBMC–-UREC co-culture. (**a**) Flow cytometry gating strategy analysis of Th1 with related histogram percentage in PBMCs (9.73 ± 4.5%) and PBMCs + URECs (4.62 ± 2.1%); and (**b**) Luminex quantification of IFN-γ secretion, results of the PBMCs + URECs condition (red) were normalized on the values of the PBMCs-alone condition (grey) set as 100%. (**c**) Flow cytometry gating strategy analysis of Treg with related histogram percentage in PBMCs (6.93 ± 1.45%) and PBMCs + URECs (12.85 ± 4.05%); and (**d**) Luminex quantification of IL-2 secretion. For flow cytometry analysis, results are expressed as mean ± SD. For cytokine quantification, results of the PBMCs + URECs condition (green) were normalized on the values of the PBMCs-alone condition (grey) set as 100%. * *p* < 0.05, n = 5 independent experiments.

After assessing the suppression of CD4^+^ and CD8^+^ cell-proliferation exerted by URECs, the following analysis focused on the effect of the co-culture on T lymphocyte subpopulations. Figure 5 shows the effect of URECs on CD4^+^ cell subsets during co-culture with PBMCs. A significant decrease in Th1 cells producing IFN-γ (CD4 ^+^IFN-γ^+^) was observed in the co-culture compared to activated PBMCs used as control (Figure 5a). The reduction in the Th1 subset was also associated with a decrease in IFN-γ secretion (Figure 5b). Among CD4^+^ cells, an increase in the percentage of Treg cells (CD4 ^+^CD25^+^Foxp3^+^) was observed in the co-culture condition, compared to control PBMCs (Figure 5c), along with an increase in IL-2 release (Figure 5d).

### 3.6. UREC–PBMC Co-Culture Influenced the Cytokine Microenvironment

**Figure 6:**
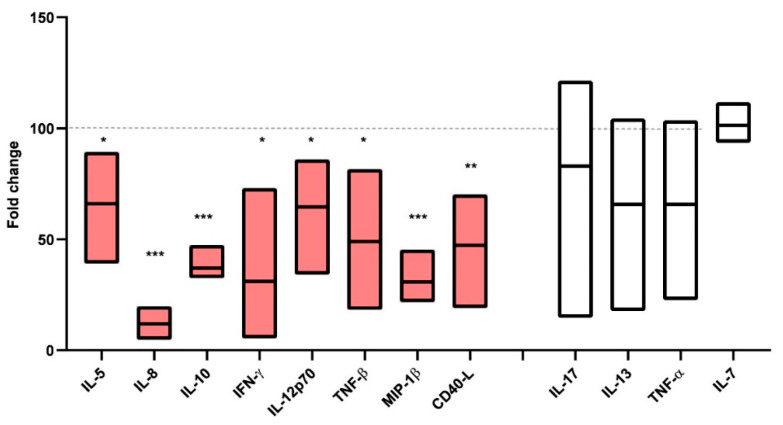
Secretion profile of PBMCs after co-culture with URECs. Histograms represent mean ± SD for each cytokine: IL-5 (66.06 ± 20.6); IL10 (37.05 ± 7); IFN-γ (31.1 ± 36.6); IL12p70 (64.75 ± 25.77); TNF-β (49 ± 31.74); MIP-1β (30.83 ± 12.68); CD40-L (47.35 ± 21.14); IL-17 (83.02 ± 48.8); IL-13 (65.75 ± 36.44); TNF-α (65.74 ± 37.5); IL-7 (101.3 ± 7.06). Results obtained after PBMC–-UREC co-culture were normalized in control PBMCs (100, dashed line). * *p* < 0.05, ** *p* < 0.01, *** *p* < 0.001, n = 5 independent experiments. Red bars were used for cytokines which decreased during co-culture, white bars for cytokines that remained stable. Abbreviations: Interleukin (IL), Interferon (IFN), Macrophage Inflammatory Protein (MIP), Tumour Necrosis Factor (TNF), CD40-Ligand (CD40-L).

The analysis of the supernatants collected after the in vitro culture of activated PBMCs with or without URECs revealed that the co-culture with urine-derived cells widely influenced the secretion profile of PBMCs. Figure 6 shows the panel of cytokines measured with Luminex in the supernatants of the previously described culture conditions. For each cytokine, results obtained from the PBMCs + URECs supernatants were normalized on the value detected in activated PBMCs.

A decrease in a wide panel of cytokines involved in the immune response and inflammation, including IL-5, IL-8, IL10, IFN-γ, IL12p70, Tumour Necrosis Factor-β (TNF-β), Macrophage inflammatory protein 1β (MIP-1β), and CD40 ligand (CD40-L), was observed during the co-culture. No significant modifications were observed for IL-17, IL-13, TNF-α or IL-7.

## 4. Discussion

The number of kidney transplants has grown exponentially over the years. Despite organs from living donors having shown a superior graft survival compared with the ones from deceased donors, the increasing demand of kidneys and the living organ-donation scarcity have resulted in the expansion of the donor pool, including donation after brain death (DBD) and donation after circulatory death (DCD) [21] (pp. 1836–1839).

Despite the fact DBD donation is the main current source of organs for transplantation, over the past decade, DCD donation has increased more than 10-fold.

Kidneys from DBD and especially DCD donors are highly exposed to IR injury, that inevitably takes place during the transplant procedure. The lack of O_2_ and nutrients promotes the accumulation of ischemic metabolites, kidney-damage markers and waste products, increasing the risk of AKI, DGF and rejection [22] (p. 20).

These events result in an increased cell turnover within nephron structures, especially in the glomerulus and in the proximal tubule, to replace the damaged cells with functional ones [23] (pp. 1714–1725). The result of the tissue regeneration process is an increase in the release of kidney-derived cells into the urine, together with other cell types deriving from the urinary tract [14] (p. 573).

URECs are a cell population highly voided in urine in response to stress factors, including post-transplant ischemia and reperfusion events, while a small percentage of these cells is detected in the urine of healthy patients. According to Lenzini and colleagues, URECs can be successfully cultured for up to four culture passages. They also highlighted the role of URECs in renal metabolic-disorder modelling, focusing on glycogen-storage diseases [24]. The characterization of URECs obtained from kidney transplanted patients recruited for this study confirmed the epithelial phenotype of this population, with the high expression of cytokeratin epithelial marker. The high CD13 expression also suggests that URECs obtained in the early stages after transplant was mainly derived from proximal tubules, while a small contribution of distal-tubule-derived cells (CD13−EpCAM^+^) was observed. According to previous studies, the progressive reduction in successful isolations observed after one and six months from transplant, and the lack of positive isolations from healthy donors, has confirmed that the release of URECs is higher in the earlier stages after transplant, when the regenerative events are more involved [3] (p. 1412).

As a result of the increased cell turnover, a significant amount of CD24^+^CD133^+^ kidney progenitor cells were detected during the first passages of the cell culture. The CD24^+^CD133^+^ population can be expanded in vitro without losing their phenotype and their self-renewal capacity, as well as the ability to differentiate into podocytes and tubular cells, contributing to kidney regeneration [25] (pp. 828–829). The presence of CD24^+^CD133^+^ progenitor cells in urine suggests their potential use for modelling genetic disorders and for personalized therapeutic approaches, avoiding both the risk associated with isolation from biopsies and the alteration in cell features caused by the immortalization process [26] (pp. 1961–1974). URECs also tested highly positive for mesenchymal stromal cell markers, including the CD73 surface molecule, which is known to be involved in the regulation of the immune response by converting pro-inflammatory adenosine monophosphate (AMP) into anti-inflammatory adenosine, reducing myocardial ischemia/reperfusion injury in animal models [27] (p. e006949). A percentage of URECs also expresses the tolerogenic molecule HLA-G, which is involved in the inhibition of CD4^+^ and CD8^+^ T cell proliferation, in the promotion of Treg cells and in foeto-maternal tolerance [28] (pp. 364–375). The regulation of the immune response is crucial in organ transplantation and in many immune-related disorders, new immunoregulatory strategies are needed to reduce the use of immunosuppressive drugs.

The injection of cells with immunomodulatory properties, such as mesenchymal stem cells (MSCs), is one of the most promising cell-therapy strategies nowadays.

URECs are usually poorly characterized for potential therapeutic applications, including their immunomodulatory potential. These cells may have the advantages of an easy collection, the lack of ethical issues and the high availability of samples, which are all main aspects of cell administration in therapeutic strategies and also in disease modelling. The lack of information regarding the role of HLA-G in cells obtained after kidney transplant has stimulated interest in deepening the understanding of the interaction between URECs and immune cells in vitro, setting up a co-co-culture with PBMCs from healthy donors. Here, for the first time, we demonstrated that, like other more-characterized cell populations, URECs obtained from DCD-transplanted kidneys significantly reduce the proliferation of CD4^+^ and CD8^+^ T lymphocytes. Interestingly, this inhibition of lympho-proliferation was not associated with the promotion of apoptotic mechanisms. On the contrary, URECs protected PBMCs against the onset of both early and late apoptosis. The inhibition of the apoptosis on PBMCs exerted by URECs has not been documented to date. Anti-apoptotic mechanisms have already been described in other cell populations, including mesenchymal stem cells from several tissues, and they are mostly associated with the secretion of factors such as transforming growth factor-β (TGF-β) and bone morphogenetic protein 15 (BMP15), that inhibit the activation of pro-apoptotic signals [29] (p. 270). To better understand the immunomodulatory activity of URECs, their influence on T CD4^+^ and CD8^+^ cell subsets was analysed. T CD4^+^ cell population-producing IFN-γ (Th1), commonly associated with inflammation and immune-response triggering, was significantly reduced in the co-culture, along with the reduced release of IFN-γ into the extracellular environment. Interestingly, the percentage of Treg cells increased during the co-culture, and could be associated with the expression of HLA-G observed in a subset of URECs, given its role in activating the regulatory T component and in the downregulation of the inflammatory response [30] (pp. 1–15). Additionally, an increase in IL-2, which is commonly related to both immune-response initiation and tolerance regulation, was observed [31] (pp. 79–119). The co-culture of URECs with immune cells also resulted in a significant decrease in several cytokines. Among these cytokines, some are known to be associated with the recruitment and activation of immune cells (IL-5, IL-8, MIP-1β, CD40L), while others play a role in the regulation of immune and inflammatory responses (IL-10, IFN-γ, TNF-β). It is known that cytokines and chemokines play pleiotropic roles, and their actions are strongly influenced by the surrounding microenvironment and by the target cells. A single cytokine may be secreted by different cells and may exert both pro-inflammatory and anti-inflammatory functions, generating multiple immune responses [32] (p. e2004433). For these reasons, the analysis of the mechanisms involved in the modulation of such molecules is challenging and requires deeper investigation. From the results obtained until now, it is possible to observe that the presence of a UREC monolayer significantly influenced the cytokine microenvironment in the co-culture, confirming the ability of urine-derived cells to modulate immune cells. Since this study mainly focused on the T-cell subsets, the effect of URECs on other cell populations in both the innate and the acquired immune response will be also evaluated.

## 5. Conclusions

The results obtained in this study indicate that the loss of kidney cells may represent a hallmark of the regeneration process during kidney-transplant follow-up.

The urine of transplanted patients represents a promising cell source for the evaluation of kidney-derived cell characteristics, including the stem/progenitor subset, and may represent an alternative to more invasive solid biopsies. Despite the events occurring after transplant, URECs voided in urine are a highly viable and proliferating cell population which has shown promising immunomodulatory properties, that may represent a useful tool in cell therapy strategies based on the regulation of the immune response, a fundamental aspect in the organ-transplant field.

## Figures and Tables

**Table 1:** UREC immunophenotype, list of markers analysed by flow cytometry and their main functions.

Marker	Function
Ki-67	Proliferation marker
Cytokeratin (Ck)	Epithelial marker
CD13	Proximal tubules marker
CD326 (EpCam)	Distal tubules marker
CD24	Kidney epithelial progenitor cells marker
CD133	Kidney epithelial progenitor cells marker
CD90	Mesenchymal stromal cells marker
CD73	Mesenchymal stromal cells marker
CD44	Mesenchymal stromal cells marker
CD105	Mesenchymal stromal cells marker
HLA-G	Tolerogenic molecule

**Table 2:** List of markers for the analysis of T cell subset and their main characteristics.

Marker	Function
CD4	T helper lymphocyte marker
CD8	T cytotoxic lymphocyte marker
CD25	T cell activation marker; T reg lymphocyte marker
FoxP3	T reg lymphocyte marker
IFN-γ	T helper 1 lymphocyte activation marker

## Data Availability

Data sharing is not applicable to this article.
